# Molecular Coronary Plaque Imaging Using ^18^F-Fluoride

**DOI:** 10.1161/CIRCIMAGING.118.008574

**Published:** 2019-08-06

**Authors:** Alastair J. Moss, Mhairi K. Doris, Jack P.M. Andrews, Rong Bing, Marwa Daghem, Edwin J. R. van Beek, Laura Forsyth, Anoop S.V. Shah, Michelle C. Williams, Stephanie Sellers, Jonathon Leipsic, Marc R. Dweck, Richard A. Parker, David E. Newby, Philip D. Adamson

**Affiliations:** 1 British Heart Foundation Centre for Cardiovascular Science (A.J.M., M.K.D., J.P.M.A., R.B., M.D., A.S.V.S., M.C.W., M.R.D., D.E.N., P.D.A.), University of Edinburgh, United Kingdom.; 2 Edinburgh Clinical Trials Unit (L.F., R.A.P.), University of Edinburgh, United Kingdom.; 3 Edinburgh Imaging, Queen’s Medical Research Institute University of Edinburgh, United Kingdom (E.J.R.v.B., M.C.W.).; 4 Department of Radiology, St Paul’s Hospital and University of British Columbia, Vancouver, Canada (S.S., J.L.).; 5 Christchurch Heart Institute, University of Otago, New Zealand (P.D.A.).

**Keywords:** angiography, computed tomography angiography, coronary artery disease, fluorides, myocardial infarction

## Abstract

Coronary ^18^F-fluoride positron emission tomography identifies ruptured and high-risk atherosclerotic plaque. The optimal method to identify, to quantify, and to categorize increased coronary ^18^F-fluoride uptake and determine its reproducibility has yet to be established. This study aimed to optimize the identification, quantification, categorization, and scan-rescan reproducibility of increased ^18^F-fluoride activity in coronary atherosclerotic plaque.

**Methods:**

In a prospective observational study, patients with multi-vessel coronary artery disease underwent serial ^18^F-fluoride positron emission tomography. Coronary ^18^F-fluoride activity was visually assessed, quantified, and categorized with reference to maximal tissue to background ratios. Levels of agreement for both visual and quantitative methods were determined between scans and observers.

**Results:**

Thirty patients (90% male, 20 patients with stable coronary artery disease, and 10 with recent type 1 myocardial infarction) underwent paired serial positron emission tomography-coronary computed tomography angiography imaging within an interval of 12±5 days. A mean of 3.7±1.8 ^18^F-fluoride positive plaques per patient was identified after recent acute coronary syndrome, compared with 2.4±2.3 positive plaques per patient in stable coronary artery disease. The bias in agreement in maximum tissue to background ratio measurements in visually positive plaques was low between observers (mean difference, −0.01; 95% limits of agreement, −0.32 to 0.30) or between scans (mean difference, 0.06; 95% limits of agreement, −0.49 to 0.61). Good agreement in the categorization of focal ^18^F-fluoride uptake was achieved using visual assessment alone (κ=0.66) and further improved at higher maximum tissue to background ratio values.

**Conclusions:**

Coronary ^18^F-fluoride activity is a precise and reproducible metric in the coronary vasculature. The analytical performance of ^18^F-fluoride is sufficient to assess the prognostic utility of this radiotracer as a noninvasive imaging biomarker of plaque vulnerability.

**Clinical Trial Registration:**

URL: http://www.clinicaltrials.gov. Unique identifiers: NCT02110303 and NCT02278211.

## Abstract 2

Supplemental Digital Content is available in the text.

**CLINICAL PERSPECTIVE**

Microcalcification is a key feature of coronary atherosclerosis which may precipitate plaque rupture and thrombotic occlusion. ^18^F-Fluoride positron emission tomography is a novel imaging technique that readily identifies regions of coronary microcalcification. The objective of this article was to assess the repeatability of in vivo coronary ^18^F-fluoride positron emission tomography imaging to detect and quantify regions of microcalcification. Using a standardized metric (coronary to atrial blood pool ratio, maximum tissue to background ratio), ^18^F-fluoride activity can be precisely and reproducibly measured within the coronary vasculature. The analytical performance of coronary ^18^F-fluoride activity is sufficient to determine whether this radiotracer can be used as a noninvasive imaging marker of plaque vulnerability in clinical trials.

**See Editorial by Salarian and Sadeghi**

Atherosclerotic plaque rupture is the commonest cause of acute coronary syndromes.^1^ Cardiovascular imaging modalities have focused on identifying the presence and severity of luminal stenoses to stratify the risk of plaque rupture events. However, luminal stenosis is a relatively late feature of coronary atherosclerosis, and the majority of rupture events occur at sites of nonobstructive plaque on antecedent angiography.^2–4^ As such, interest has grown in identifying adverse atherosclerotic plaque characteristics and regions of increased disease activity within the arterial wall that may better predict subsequent plaque rupture and clinical events. One promising approach has been to use combined positron emission tomography (PET) and computed tomography (CT) angiography that coregisters the detailed anatomy of the arterial wall with the in vivo biological activity of disease.^5^

To date, PET-CT has predominantly been used to evaluate the presence of inflammatory processes in large caliber arteries using the radiotracer ^18^F-fluorodeoxyglucose.^6^ However, the application of this tracer to the coronary circulation is limited because background myocardial ^18^F-fluorodeoxyglucose uptake obscures and prevents assessment of coronary plaque activity.^7^ Recent studies have found that ^18^F-sodium fluoride (^18^F-fluoride) holds major promise in identifying culprit plaques in the coronary circulation after myocardial infarction.^5,7^ Validation work in the carotid arteries indicates that ^18^F-fluoride also identifies culprit plaques after neurovascular events and preferentially binds to microcalcification, a key component of high-risk atherosclerotic plaque.^8,9^ Studies are now ongoing to assess the prospective prognostic capability of coronary ^18^F-fluoride PET and whether the signal can help guide the use of novel therapies. However, coronary ^18^F-fluoride imaging is challenging due to the small caliber of the vessels and their near-continuous motion throughout the cardiac cycle.^10,11^ Moreover, there is a lack of consensus about image analysis techniques with a number of different approaches having been used. Hence, there is a need to optimize and to standardize coronary PET imaging methodology to facilitate the widespread application of coronary molecular imaging.

To address these issues, we undertook a prospective observational clinical study in patients with stable and unstable coronary artery disease that aimed to establish a reproducible methodology for identifying, quantifying, and characterizing ^18^F-fluoride activity in the coronary arteries.

## Methods

The data supporting the findings of this study are available from the corresponding author on reasonable request, within the limits of the ethical permits of the study.

### Study Population

Participants were recruited within prespecified reproducibility substudies of the ongoing DIAMOND (Dual antiplatelet therapy to Inhibit coronary Atherosclerosis and Myocardial injury in patients with Necrotic high-risk coronary plaque Disease) and PRE^18^FFIR (Prediction of Recurrent Events with ^18^F-Fluoride to Identify Ruptured and High-risk Coronary Artery Plaques in Patients with Myocardial Infarction) trials. Inclusion required the presence of multi-vessel coronary artery disease on invasive angiography, either after recent myocardial infarction (PRE^18^FFIR), or in the context of stable coronary artery disease (DIAMOND). Exclusion criteria included inability to receive iodinated contrast, renal impairment (estimated glomerular filtration rate ≤30 mL/min per 1.73 m^2^) or women of child-bearing potential. The study was approved by the local institutional review board, the Scottish Research Ethics Committee (REC reference: 14/SS/0089 and 15/SS/0203), and the United Kingdom Administration of Radiation Substances Advisory Committee. It was performed in accordance with the Declaration of Helsinki. All patients provided written informed consent before any study procedures.

### ^18^F-Fluoride PET and Coronary CT Angiography

All patients underwent ^18^F-fluoride PET-CT and coronary CT angiography scanning on 2 occasions 2 weeks apart using the same protocol. Patients were administered 50 to 100 mg oral metoprolol if their resting heart rate was >65 bpm before the intravenous administration of 250 MBq ^18^F-fluoride. After 60 minutes, patients were imaged with a hybrid PET-CT scanner (64-multidetector Biograph mCT, Siemens Medical Systems, Erlangen, Germany). Attenuation correction CT scans were performed in held expiration before the acquisition of electrocardiographic-gated list-mode PET data using a single 30-minute bed position centered on the heart. Finally, an electrocardiographic-gated coronary CT angiogram (CCTA) was performed in mid-diastole during held expiration. All patients received sublingual glyceryl trinitrate before CCTA.

### Image Analysis

Electrocardiographic-gated PET images were reconstructed in diastole (50%–75% of the R-R interval, 2 iterations, 21 subsets Siemens Ultra-HD algorithm) and fused with contrast-enhanced CCTA. Analysis of the CT images was performed using dedicated software (Vitrea Advanced, Toshiba Systems) with multi-planar reformatting for plaque analysis used as necessary. Coronary arteries with a diameter ≥2 mm were assessed according to the 18-segment Society of Cardiac CT model.^12^ Qualitative and semi-quantitative analysis of the PET images from all 60 scans were performed independently by trained observers using an OsiriX workstation (OsiriX version 3.5.1 64-bit; OsiriX Imaging Software, Geneva, Switzerland).

### Identification of Coronary ^18^F-Fluoride Uptake

Coregistration of PET and CCTA images was undertaken to aid image interpretation in a 2 stage process. First, ^18^F-fluoride blood pool activity on the PET scan was aligned with contrast-enhanced CCTA images of the cardiac chambers in 3 dimensions using axial, sagittal, and coronal views of the heart. This approach is made possible by the increased blood pool activity of ^18^F-fluoride in comparison to the myocardium, allowing the contours of the cardiac chambers to be determined on the PET images. Second, axial, coronal, and sagittal views were again interrogated in 3 dimensions to ensure optimal alignment of any tracer uptake in the aortic valve, aortic root, and the inner curve of the ascending aorta with the contrast CCTA (Figure I in the Data Supplement).

Visual assessment for increased coronary ^18^F-fluoride activity was performed on both a per-patient–level and per-segment basis. For a signal to be colocalized to the coronary artery, a coronary atherosclerotic plaque had to be present on the CCTA, and the increased pattern of radiotracer had to arise from the coronary artery and follow its course over >5 mm in 3 dimensions on orthogonal views. Care was taken to exclude ^18^F-fluoride activity arising from adjacent structures, such as the aortic valve, mitral valve annulus, left atrial appendage, and the pulmonary artery.

### Quantification of Coronary ^18^F-Fluoride Uptake

Semi-quantitative PET analysis was undertaken for all proximal coronary segments in addition to any atherosclerotic segment with focal ^18^F-fluoride activity as described above. First, maximum standardized uptake values (SUV_MAX_) were measured within regions of interest drawn around these areas. Due to the difficulties of drawing reproducible regions of interest around the perimeter of coronary segments, mean SUV values were not recorded.

SUV_MAX_ values were corrected for background activity using several different methods. First, correction was made for uptake in a referent proximal coronary plaque with no evidence of increased ^18^F-fluoride activity.^5,7^ Second, correction was made for blood pool activity using elliptical regions of interest drawn within the brachiocephalic vein, superior vena cava, and all 4 cardiac chambers with mean standardized uptake values (SUV_MEAN_) recorded for each region. To calculate coronary target-to-background ratios (TBR), coronary SUV_MAX_ was divided by these background measures providing TBR_REFERENT_ and TBR_MAX_ values, respectively.

### Categorization of Coronary ^18^F-Fluoride Uptake

We next evaluated whether tracer quantification could be used to improve on the visual criteria described above for assessing coronary ^18^F-fluoride activity. ^18^F-Fluoride activity in plaques meeting visual criteria for positivity was quantified and use to categorize plaques into the following groups: uptake below (TBR <0.9), similar to (TBR 0.9–1.1), and above (TBR >1.1) blood pool activity. This categorization was then applied to both patients with recent acute coronary syndromes and stable coronary artery disease to assess the frequency of ^18^F-fluoride active lesions with the different thresholds. Discrepancies in reporting were resolved by consensus adjudication (2 out of 3 blinded observers) of the presence of focal coronary ^18^F-fluoride activity.

### Reproducibility of Coronary ^18^F-Fluoride Uptake

Repeat anonymized scans for all 30 patients were presented to 3 experienced observers in random order. Each observer performed scan analysis independent of the other readers. First, they determined the presence of coronary plaques with increased visual activity using the criteria for visual identification described above. Second, they quantified activity in the coronary plaques which were referenced to background activity in the referent plaque, right atrium, left atrium, right ventricle, left ventricle, superior vena cava, and brachiocephalic vein. Coefficients of variation for each of these background measurements were then calculated. Subsequently, coefficients of variation were calculated for each metric of coronary ^18^F-fluoride activity (eg, SUV_MAX_, TBR_MAX_, TBR_REFERRENT,_ etc). Finally, we assessed the agreement of these metrics across serial scans using visual categorization in isolation, and in combination with a low semi-quantitative threshold (TBR >0.9) or a high semi-quantitative threshold (TBR >1.1).

### Statistical Analysis

Continuous variables are reported as mean±SDor median and interquartile range. Categorical variables are reported as absolute number and percentages. Paired *t* tests were used to determine statistical significance for comparisons of means with normal distribution. Consensus observer agreement (2 out of 3 observers) of coronary segment tracer localization was used to adjudicate positive focal tracer uptake, and the presence of a single positive segment was sufficient for a patient-level diagnosis of ^18^F-fluoride positivity. Limits of agreement analysis were conducted to assess scan-rescan and interobserver repeatability for all coronary segments and visually positive coronary plaques using TBR_MAX_ adjusted for the left atrial blood pool. After the mixed-effects limits of agreement methodology of Parker et al,^13^ a mixed-effects model was fitted to the TBR_MAX_ between-scan paired differences with segment included as a random effect nested within patient, adjusting for study (DIAMOND or PRE^18^FFIR), and observer as fixed effects. The mixed model for the TBR_MAX_ between-observer paired differences was similar but with scan number and observer comparison as fixed effects instead of observer. In both cases, the mean bias was separately estimated based on a model only including the nested random effects of segment within patient.^13^ Variance components concordance correlation coefficients (CCC) were computed as an additional measure of scan-rescan and inter-rater agreement using mixed-effects methodology suitable for repeated measures data.^14^ Specifically, we fitted a mixed-effects model to the raw outcome data including nested random effects of segment within patient and adjusting for observer, study (DIAMOND or PRE^18^FFIR), scan (first or second), and the global mean as fixed effects. For assessment of scan-rescan agreement we included the scan-rescan variance in the denominator of the repeated measures CCC formula, whereas for interobserver agreement we included the between-observer variance in the denominator instead. Note that the CCC is strongly dependent on the between-segment and between-patient variability. Pan et al^15^ suggest that the CCC variance components method calculated using mixed-effects modeling could also be applied to binary outcome data, and concludes that the method works well when the proportions are not too extreme. Therefore, the same CCC method was used to measure agreement of the binary visual uptake outcome to produce a kappa-type statistic κ. The κ values were interpreted as follows: poor ≤0.20, fair 0.21 to 0.4, moderate 0.41 to 0.60, good 0.61 to 0.80, and very good ≥0.81. A nonparametric bootstrap method was used to compute 95% bias-corrected percentile CI for the CCC estimates. Statistical analysis was performed using R version 3.5.3 (R Foundation for Statistical Computing, Vienna, Austria). Statistical significance was taken as *P*<0.05.

## Results

Thirty patients (90% male, 20 patients with stable coronary artery disease, and 10 with recent type 1 myocardial infarction) underwent serial PET-CCTA imaging within 12±5 days. Cardiovascular risk factors were common, and 28 (93.3%) patients had undergone prior coronary revascularisation (22 percutaneous coronary intervention and 9 coronary artery bypass grafting; Table 1).

**Table 1.**
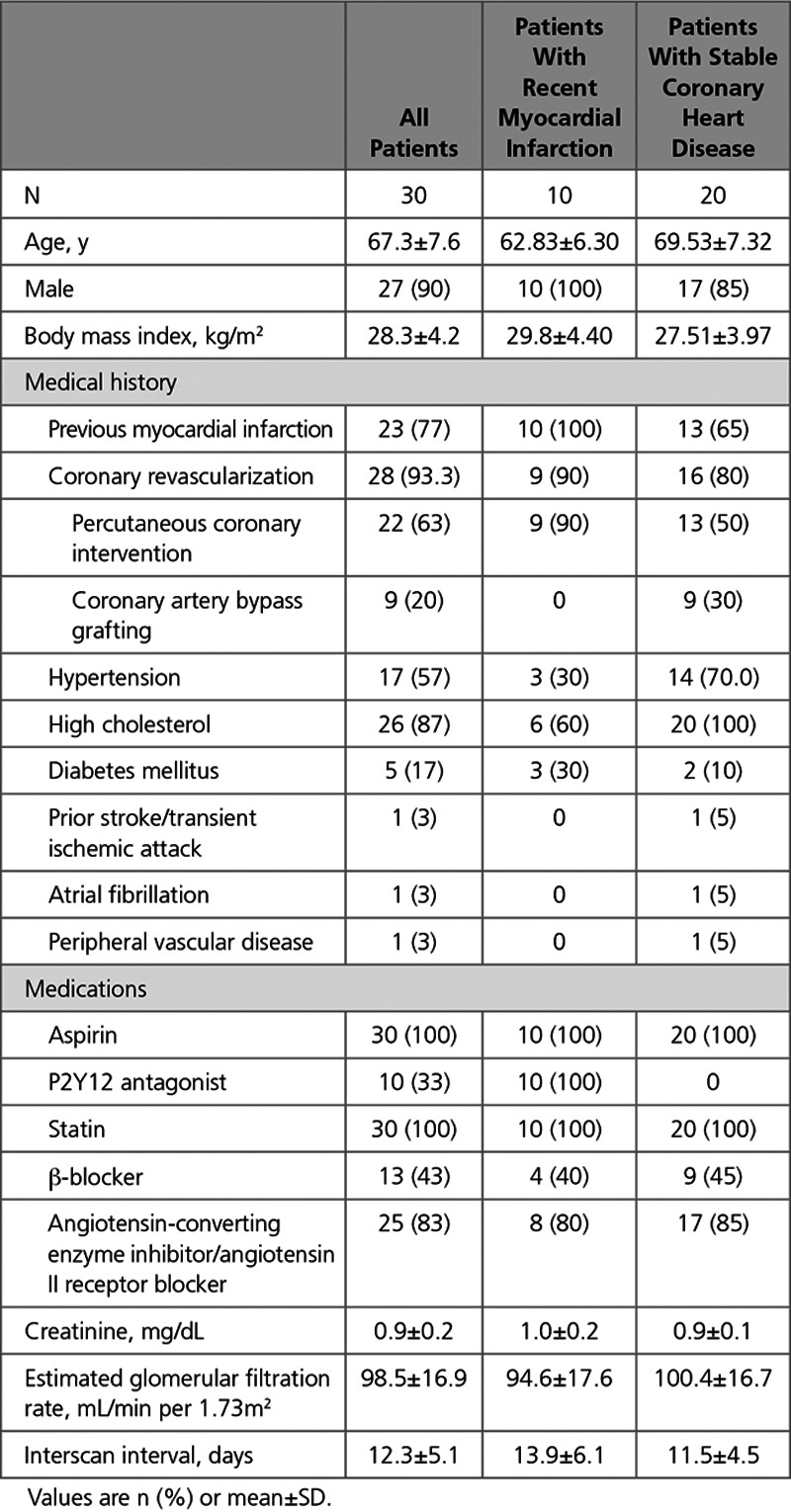
Baseline Characteristics of Study Population

### Blood Pool ^18^F-Fluoride Activity

**Figure 1.**
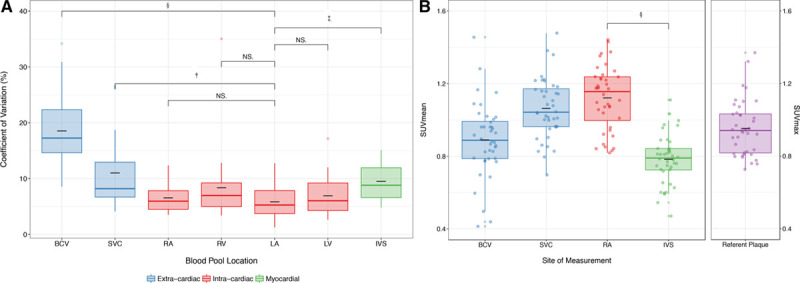
**Background blood pool and cardiac ^18^F-fluoride activity.** The mean standardized uptake value (SUV_MEAN_; g/mL) in each region was compared by 3 observers across 2 scans. **A**, Box-plot of the median and the interquartile range of coefficients of variation for each region (black line, mean). There was no difference in the coefficients of variation of ^18^F-fluoride SUV_MEAN_ within intracardiac chambers (red), but there were increased coefficients of variation using systemic venous blood pool measurement (*P*<0.001) and interventricular septal myocardium (*P*<0.001) compared with measurement of SUV_MEAN_^18^F-fluoride activity in the left atrium. **B**, Box-plot of the median and the interquartile range of mean standardized uptake values for brachiocephalic, superior vena cava, right atrium, and interventricular septum (black line, mean). Note the low myocardial and background coronary arterial ^18^F-fluoride uptake compared with blood pool in the right atrium. BCV indicates brachiocephalic vein; IVS, interventricular septum; LA, left atrium; LV, left ventricle; NS, not-significant; RA, right atrium; RV, right ventricle; and SVC, superior vena cava. *P≤0.05; †*P*≤0.01; ‡*P*≤0.001; and §*P*≤0.0001.

^18^F-Fluoride blood pool activity was consistently higher in all 4 cardiac chambers than in the myocardium (blood pool SUV_MEAN_ 1.14 versus interventricular septum SUV_MEAN_ 0.80; Figure 1, FigureII in the Data Supplement). This facilitated accurate manual coregistration of the PET and CCTA data sets as described above (Figure I in the Data Supplement). Background measurements in the referent plaque, brachiocephalic vein, and the superior vena cava were consistently lower than cardiac blood pool with high coefficients of variation. The regions with the least variability in serial measurements of background activity (SUV_MEAN_) were the cardiac atria with coefficients of variation in the left and right atria of 5.9±2.8% and 6.5±2.5%, respectively (Figure 1, Table I in the Data Supplement).

### Identification of Coronary ^18^F-Fluoride Uptake

**Figure 2.**
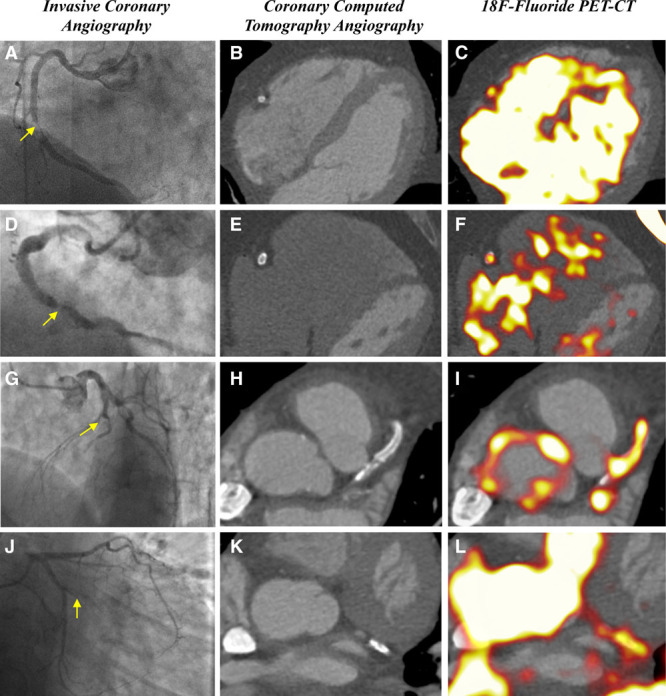
**Culprit plaque ^18^F-fluoride activity on positron emission tomography-coronary computed tomography angiography (PET-CT).** After acute myocardial infarction, culprit plaque ^18^F-fluoride activity can be measured in the right coronary artery (**A**–**F**), left anterior descending artery (**G**–**I**), and atrioventricular circumflex artery (**J**–**L**).

Low myocardial ^18^F-fluoride uptake enabled visual separation between areas of focal ^18^F-fluoride binding in the coronary arteries and background blood pool activity in the cardiac chambers. After accurate coregistration, these factors facilitated the identification of radiotracer originating from the coronary arteries even at levels of intensity similar to background blood pool activity that would otherwise not be discernible (Figure 2). Using this method, focal coronary ^18^F-fluoride activity of variable intensity could be visually identified in all culprit plaques (n=10) in patients with recent type 1 myocardial infarction.

### Quantification of Coronary ^18^F-Fluoride Uptake

**Figure 3.**
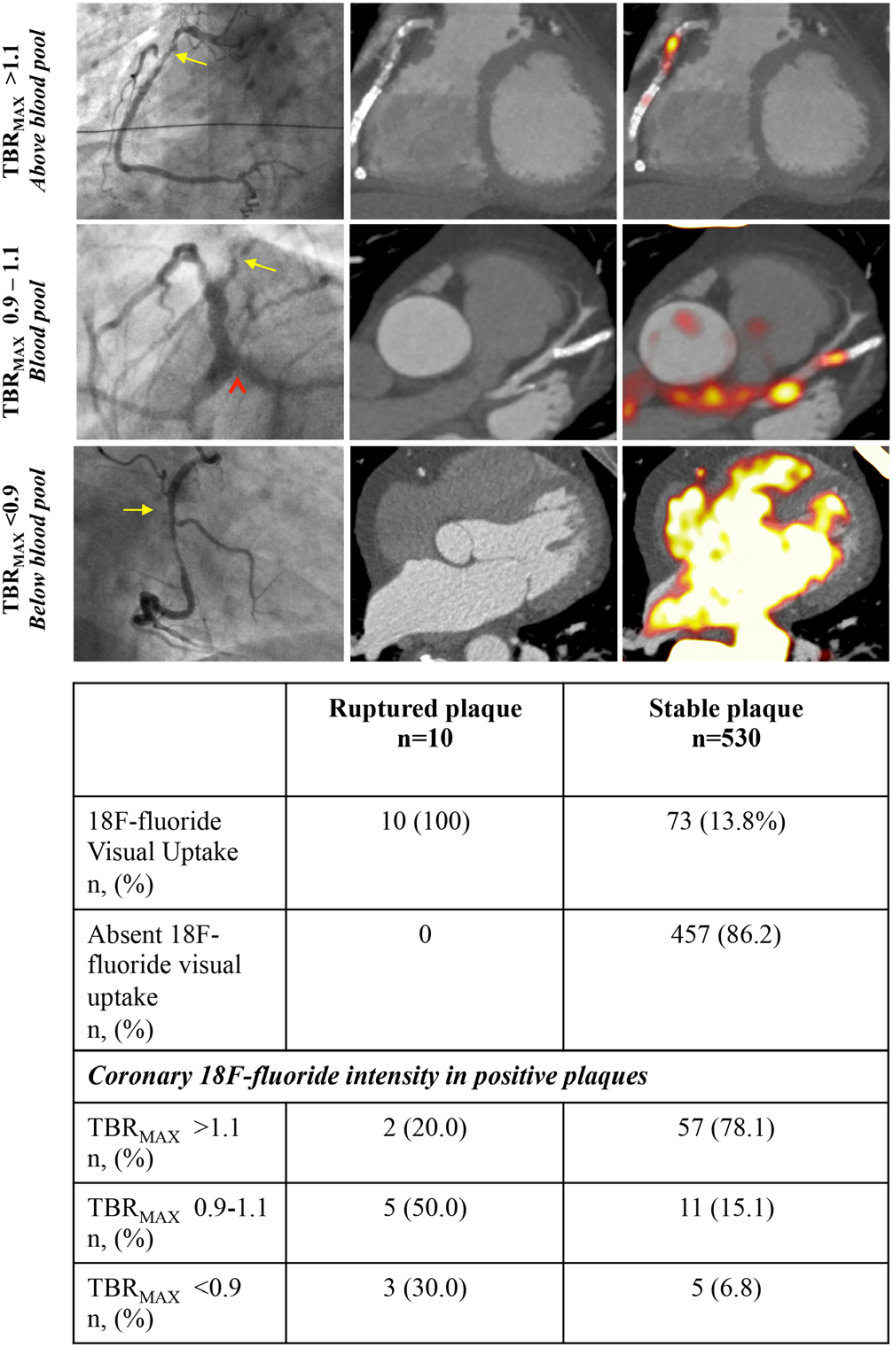
**Quantification of coronary ^18^F-fluoride activity.** Visual identification of coronary ^18^F-fluoride activity was present in all ruptured plaques and 13.8% of stable coronary segments. The signal intensity of ^18^F-fluoride activity in coronary plaque was assessed both visually and semi-quantitatively by referencing to atrial blood pool activity (maximum target-to-background ratio [TBR_MAX_]). Activity in coronary plaques was categorized into activity above blood pool (TBR_MAX_ >1.1), at or around blood pool (TBR_MAX_ 0.9–1.1), or below blood pool (TBR_MAX_ <0.9). Higher intensity signals were observed in stable segments compared with ruptured plaques.

Quantification of coronary ^18^F-fluoride activity within the plaque of interest, standardized according to a proximal nondiseased coronary artery segment (TBR_REFERENT_) demonstrated a high degree of variability on repeated measurements. The coefficients of variation reduced in a stepwise manner when using the brachiocephalic vein and superior vena cava as the sites of measurement for blood pool activity and were lowest for target-to-background ratios determined after standardization for intracardiac blood pool activity using either the left or right atria (*P*<0.05 for all comparisons, Figure III in the Data Supplement). Therefore, subsequent analyses used TBR_MAX_ values standardized according to the atrial blood pool, with ^18^F-fluoride originating in the coronary arteries categorized into levels below (TBR_MAX_ <0.9), similar to (TBR_MAX_ 0.9–1.1) and above (TBR_MAX_ >1.1) atrial blood pool activity (Figure 3).

### Categorization of Coronary ^18^F-Fluoride Uptake

Using atrial blood pool as a point of reference allowed comparison of coronary ^18^F-fluoride TBR_MAX_ between patients with recent acute coronary syndromes and stable coronary artery disease. At the patient level, there were 3.7±1.8 positive plaques in patients with recent acute coronary syndrome compared with 2.4±2.3 positive plaques in patients with stable disease. Among patients with recent myocardial infarction, 100% (n=10/10) of culprit plaque segments met the criteria for visual uptake compared with 13.7% (n=73/530) of all segments not associated with plaque rupture. Visual assessment of coronary segment positivity achieved good interobserver reliability (κ=0.66, 95% CI, 0.63–0.70). Minor discrepancies between reporters occurred in regions adjacent to high background activity including the right atrial blood pool, pulmonary artery and mitral valve annulus that limited assessment in regions of the right, left anterior descending artery, and atrioventricular circumflex arteries, respectively. Agreement increased among coronary segments with higher TBR_MAX_ values and the application of a semi-quantitative method of determining ^18^F-fluoride positivity, requiring both visual evidence of tracer localization to a coronary region of interest and TBR_MAX_ above the prespecified intensity thresholds achieved good scan-rescan agreement at both predefined cut points (TBR_MAX_ >0.9, κ=0.72; 95% CI, 0.67–0.77; TBR_MAX_ >1.1 κ=0.77; 95% CI, 0.71–0.83; Figure 3 and Figure IV in the Data Supplement). When applied to patients with recent myocardial infarction, the use of TBR_MAX_ thresholds resulted in fewer culprit plaques being classified as positive for focal ^18^F-fluoride uptake (n=7/10 [70%] at TBR_MAX_ ≥0.9; n=2/10 [20%] at TBR_MAX_ >1.1).

### Reproducibility of Coronary ^18^F-Fluoride Uptake

**Figure 4.**
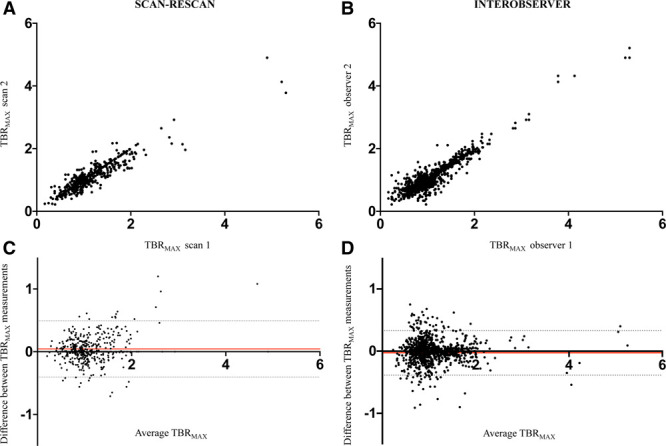
**Bland-Altman plots of coronary ^18^F-fluoride activity.** Correlation and Bland-Altman plots for scan-rescan reproducibility for coronary plaques at different levels of coronary ^18^F-fluoride activity (TBR_MAX_; **A** and **C**, respectively). Correlation and Bland-Altman plots for interobserver reproducibility between 2 observers at different levels of ^18^F-fluoride activity (TBR_MAX_; **B** and **D**, respectively). The shaded circles represent individual observations whilst the red and grey hatched lines represent the mean difference and 95% mixed-effects limits of agreement, respectively.

There were no differences in scan variables between the serial scans (Table II in the Data Supplement). Patients with stable coronary artery disease had an increased dose-length product than patients with recent MI due to the wider field of view on coronary CT angiography required for assessing the origin of the left internal mammary artery in cases of previous coronary artery bypass grafting. In all measured coronary segments (n=519), there was a mean bias of −0.03 between observers (95% limits of agreement −0.39 to 0.33) and a mean bias of 0.04 between scans (95% limits of agreement −0.41 to 0.49; Table 2 and Figure 4). In visually positive plaques, mixed-effects biases were low between observers (mean bias −0.01; 95% limits of agreement −0.32 to 0.30) and between scans (mean bias 0.06, 95% limits of agreement −0.49 to 0.61; Table 2). There was very good concordance between observers (concordance correlation coefficient 0.88; 95% CI, 0.85 to 0.90) and between scans (concordance correlation coefficient 0.86; 95% CI, 0.83 to 0.89). At the level of the patient, there was 100% agreement between the repeat scans on whether a patient demonstrated any increased coronary ^18^F-fluoride activity in at least one plaque. At the per-segment level, there was excellent agreement (92.4%) between scans using the visual assessment alone, although further improvements in agreement were achieved by applying the TBR_MAX_ thresholds (93.0% agreement rate TBR_MAX_ ≥0.9; 94.2% agreement rate TBR_MAX_ >1.1; Figure IV in the Data Supplement).

**Table 2.**
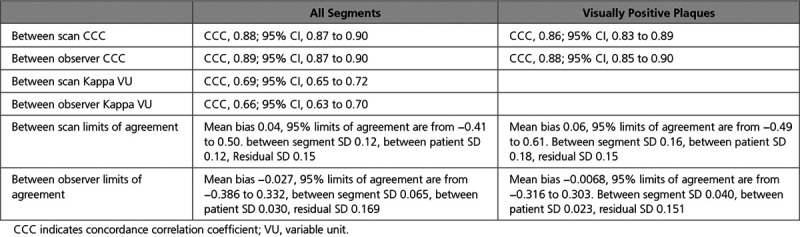
Mixed-Effects Limits of Agreement Analysis Between Scans and Observers for All Segments and Visually Positive Plaques

## Discussion

This study describes an optimized methodology for assessing coronary ^18^F-fluoride activity in vivo. Using this approach, there is good agreement in the identification of plaques with increased coronary ^18^F-fluoride uptake between observers and on different scans. The tissue (SUV_MAX_) to background (left atrial SUV_MEAN_) ratio (TBR_MAX_) has a low bias between observers and between scans. At TBR_MAX_ thresholds >0.9, there is low sampling variability and high interobserver agreement in the adjudication of coronary ^18^F-fluoride activity. ^18^F-Fluoride demonstrates optimal characteristics for coronary plaque molecular imaging, namely, low myocardial and background coronary artery activity which allows regions of increased coronary ^18^F-fluoride uptake to be more readily detected at low levels of intensity. This technique has direct application to ongoing clinical trials using the identification of atherosclerotic plaque mineralization to predict coronary events and improve the stratification of emerging therapeutic interventions.

Our study has a number of notable strengths. First, this is the largest prospective study to investigate the scan-rescan reproducibility of coronary imaging with repeated ^18^F-fluoride PET-CT scans. Second, the study population comprising 30 patients with established, multi-vessel coronary disease represents a high-risk cohort for whom imaging biomarkers have genuine potential to improve prognostic stratification and guide therapeutic interventions. Third, we directly compared several proposed methods for quantifying focal tracer activity within coronary atherosclerotic lesions and determined the interobserver and scan-rescan agreement for these metrics. Lastly, we derived a standardized semi-quantitative approach to image analysis that allows consistency in the reporting of diagnostic findings.

For an imaging biomarker to have clinical utility, it should demonstrate precise and reproducible analytical performance.^16^ Specifically, precision refers to the closeness of agreement between independent observations under stipulated conditions (free from random error) and reproducibility reflects to the differences observed over separate scan acquisitions. The methodology of coronary ^18^F-fluoride assessment presented here has been optimized to define a precise measure of background activity (cardiac atria) that yields a precise measure of coronary activity (TBR_MAX_) across serial scan acquisitions. Demonstrating the reliability of this metric is an important step towards implementing coronary PET imaging in clinical practice and prospective research trials. We suggest such studies apply similar acquisition and image analysis protocols to that described here, and continue to explore the clinical significance of such measurements.

To discriminate ^18^F-fluoride uptake from levels of background signal, focal coronary artery activity has previously been referenced to proximal nondiseased coronary artery segments (TBR_REFERENT_).^5,7^ Although this practice differs from noncoronary ^18^F-fluoride PET imaging, it has been considered more appropriate to reference to a similar sized structure rather than a large blood pool region to avoid artificially low TBR values related to partial volume effects. However, we found this metric to have a high degree of variability on serial testing, due to inconsistency in measurements in the referent plaque. This likely reflects the inherent difficulty in reproducibly measuring a coronary segment without visual ^18^F-fluoride activity compared with the much easier agreement between observers when drawing a region of interest around a focal site of tracer uptake. Of note, this study identified ^18^F-fluoride activity within distal and side branch vessels that may be more susceptible to partial volume averaging effects. In these regions, activity may spill out into surrounding structures, thereby decreasing counts in plaques which would otherwise have increased activity. Similarly, coronary segments in close proximity to calcified tissue in the aortic and mitral annulus may be subjected to spill-in of ^18^F-fluoride activity in regions that would otherwise have low activity. These small changes in localization of maximal activity explain the variation observed when measuring structures below the field-width half maximum of ^18^F-fluoride (6 mm). Future work using an increased delay between injection and acquisition^17^ optimizing the reconstruction algorithm and motion-correcting the list mode data^18^ may improve quantitative reproducibility. Thus, quantification of activity should only be undertaken in plaques that meet the visual criteria stipulated in the methodology.

Our optimized methodology for estimating background blood pool activity also differs from previous ^18^F-fluorodeoxyglucose cardiovascular PET studies. Here, background blood pool activity is measured within the large venous capacitance vessels, such as the superior vena cava.^19,20^ This is preferred over measurements from within the cardiac chambers because ^18^F-fluorodeoxyglucose is taken up by viable myocardium and blood pool activity can be contaminated by signal overspill from the myocardium. In contrast, there is very low myocardial uptake of ^18^F-fluoride and thus estimating atrial blood pool activity is not susceptible to this confounding issue. The superior vena cava can be a relatively small compressed structure sensitive to sampling error and partial volume effects whereas the atria present larger spherical volumes of activity that can be more readily and reliably sampled, providing a measurement with higher precision and reproducibility. Thus for tracers with a low background myocardial uptake, background blood pool activity would appear to be best measured in the cardiac atria. Consequently, although standardization of coronary activity with reference to the atrial blood pool (TBR_MAX_) resulted in numerically lower values than when standardized according to either a referent coronary segment or an extracardiac blood pool, the coefficients of variation were much reduced. Indeed, because the referent plaque uptake is lower (0.7–0.8 of that in the atrial blood pool), our previous TBR_MAX_ threshold of 1.25 equates to a TBR_MAX_ threshold 0.9 to 1.0 when using the atrial blood pool activity. This finding has important implications for future studies that seek to measure change in tracer signal over serial scans, either to explore the natural history of plaque vulnerability or to use this metric as a surrogate for therapeutic response.

While quantitative measurements of ^18^F-fluoride activity have particular relevance to serial imaging studies, it is not clear that the numeric value of the TBR_MAX_ has direct correlation with plaque vulnerability. Indeed, we found that among patients with acute myocardial infarction, the culprit plaques demonstrated proportionally lower TBR_MAX_ values than visually positive plaques in patients with clinically stable disease. Although somewhat counterintuitive, this finding is consistent with our previous report,^7^ and the relationship between TBR_MAX_ and risk of future clinical events remains to be determined in large-scale prospective trials, such as the PRE^18^FFIR trial. Indeed, it should be remembered that ^18^F-fluoride uptake correlates with microcalcification and is a marker of calcification activity.^7^ While it identifies high-risk plaque with a necrotic core, the magnitude of uptake may reflect different stages of the calcification process, and it may be the presence rather than the magnitude of uptake that is most important. For example, high ^18^F-fluoride uptake may represent advanced calcification activity with rapidly developing macrocalcification, which is stabilizing the plaque and making it less prone to rupture. Conversely, relatively lower levels of ^18^F-fluoride uptake may represent the immediate response to a recently developed unstable plaque with a necrotic core that has yet to develop demonstrable macrocalcification and has only nascent calcification activity.

We have demonstrated good reliability for visual assessment alone in the identification of culprit plaques after recent acute coronary syndrome. Perhaps unsurprisingly, the interobserver and scan-rescan agreement for the classification of ^18^F-fluoride positivity within a coronary segment increased inline with the TBR_MAX_ and was greatest for coronary segments with maximum standardized uptake values >1.1×the blood pool SUV_MEAN_. In this regard, a recent cohort study has demonstrated that coronary TBR_MAX_ ≥1.28 stratified individuals at increased risk of plaque rupture.^21^ While this higher patient-level threshold identified individuals at risk of late coronary revascularisation, at the plaque-level 45% of culprit plaques (n=5/11) did not meet this threshold.^21^ This observation is inkeeping with our finding that culprit plaque ^18^F-fluoride activity, while readily identifiable, is often at a lower intensity than blood pool which future studies may need to account for. The identification of low-intensity ^18^F-fluoride activity needs to be prospectively evaluated in future studies of repeated imaging following an acute coronary syndrome.

This study has a number of limitations. It was undertaken in a single center with extensive experience in cardiac ^18^F-fluoride PET-CCTA imaging and scans were performed using a single PET-CCTA scanning system. It is important to acknowledge that many operator and system-dependent variables can influence final image quality, and these will necessitate careful consideration when applying our findings in different settings. A number of limitations of our study are inherent to coronary PET imaging, and include the challenges of nonspecific binding, cardiac motion, partial voluming, and relatively low signal-to-noise ratios. We chose to use ^18^F-fluoride as our PET tracer as there is low physiological activity within the myocardium, reducing the chance for spill-in activity. Furthermore, we have previously established that the problem of cardiac motion can be overcome through electrocardiographic-gating in a similar manner to that employed in coronary CT angiography.^22^ Although restricting the analysis of the PET signal to one-quarter of the cardiac cycle reduces the signal-to-noise ratio, we have now compensated for this by increasing tracer dose, increasing scan duration, and applying advanced image reconstruction algorithms including time-of-flight reconstruction with point spread function modeling. In combining these incremental gains, we have derived a semi-quantitative measure of plaque pathophysiology that is highly reproducible on interval scanning and is well placed for observing dynamic changes in activity over time.

In conclusion, ^18^F-fluoride PET-CCTA imaging is highly reproducible over repeated scans and between multiple observers. A semi-quantitative approach achieves repeatable metrics than can now be assessed to determine their clinical significance. This approach to image acquisition and analysis provides strong support for the ongoing clinical studies investigating the value of ^18^F-fluoride PET-CCTA to inform cardiovascular prognostic assessment and guide novel therapeutic strategies.

## Acknowledgments

The authors acknowledge the contributions of Mrs Audrey Kuchnowski and the staff at the Wellcome Trust Clinical Research Facility and Edinburgh Imaging Facility at the Royal Infirmary of Edinburgh.

## Sources of Funding

Drs Moss, Forsyth, and Newby are supported by a Wellcome Trust Senior Investigator Award (WT103782AIA). Dr Newby (CH/09/002, RG/16/10/32375, RE/13/3/30183), Dr Doris (FS/17/79/33226), Dr Andrews (FS/17/51/33096), and Dr Dweck (FS/14/78/31020) are supported by the British Heart Foundation. Dr Williams is supported by The Chief Scientist Office of the Scottish Government Health and Social Care Directorates (PCL/17/04). Dr Shah is supported by an Intermediate Clinical Research Fellowship from the British Heart Foundation (FS/19/17/34172). Dr van Beek is supported by the Scottish Imaging Network. A Platform of Scientific Excellence (SINAPSE). The Edinburgh Clinical Research Facilities and Edinburgh Imaging facility are supported by the National Health Service Research Scotland (NRS) through National Health Service Lothian Health Board.

## Disclosures

None.

## Supplementary Material
