# Supplementary material for: Molecular Coronary Plaque Imaging Using 18F-Fluoride
Source: Circ Cardiovasc Imaging. 2019 Aug 6;12(8):e008574. doi: 10.1161/CIRCIMAGING.118.008574 (PMC7668410; doi:10.1161/CIRCIMAGING.118.008574)
Supplement: Supplementary file 1 [file hci-12-e008574-s001.docx]

**Supplementary Figure 1.**

**Co-registration of 18F-fluoride positron emission tomography with contrast-enhanced computed tomography.**

Patient and cardiac motion between the acquisition of positron emission tomography (PET) and coronary computed tomography angiography (CCTA) datasets can be enhanced by co-alignment of diastolic phase electrocardiogram-gated PET reconstructions (50-75% of cardiac cycle) with the diastolic phase of CCTA. 18F-Fluoride activity in cardiac chambers facilitates accurate 3-dimension co-alignment in axial and coronal views. At low levels of activity, 18F-fluoride is predominantly restricted to the intravascular compartment with minimal activity observed in the myocardium. 18F-Fluoride activity that co-localises to the inner and outer curvature of the ascending aorta and aortic valve can be used as markers for co-registration. 18F-Fluoride activity in coronary arteries is referenced to blood pool activity allowing coronary 18F-fluoride activity to be differentiated from the surrounding structures in multiplanar views.


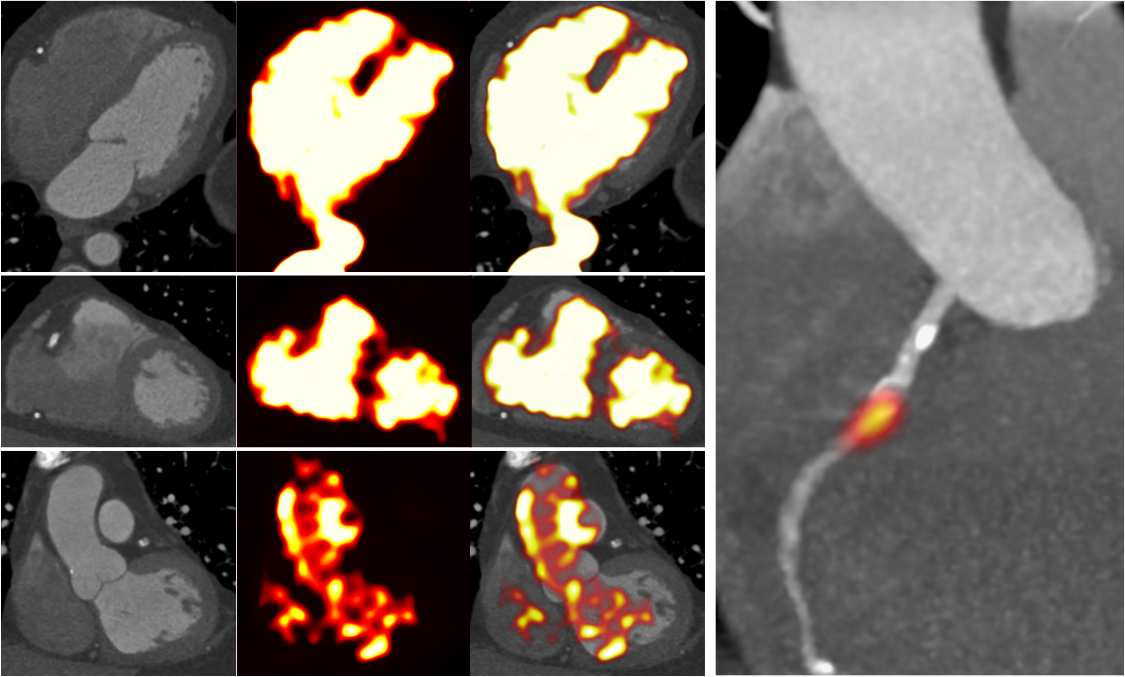


**Supplementary Figure 2.**

**Background cardiac 18F-fluoride activity**

The mean standardised uptake value SUV­_MEAN­_ (g/mL) in each location in all patients with coronary artery disease.

The horizontal bar inside each box is the median, the lower and upper hinges correspond to the first and third quartiles (the 25th and 75th percentiles). The upper and lower whiskers extend from the hinge to the largest or smallest value no further than 1.5 times the interquartile range from the hinge. The short black horizontal line corresponds to the mean value.

BCV, brachiocephalic vein, IVS, interventricular septum, LA, left atrium, LV, left ventricle, RA, right atrium, RV, right ventricle, SVC, superior vena cava.


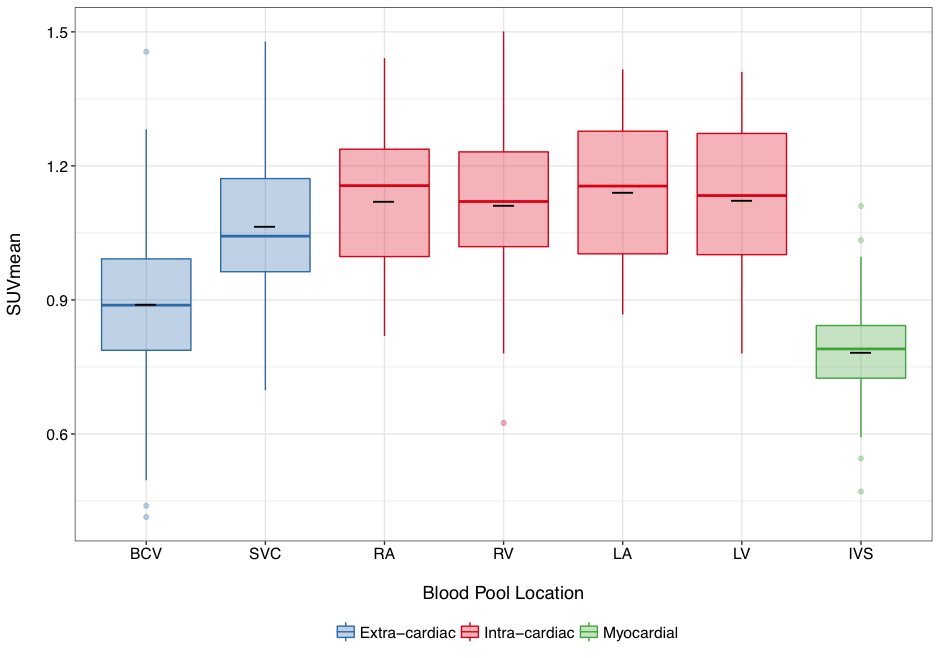
**Supplementary Figure 3.**

**Co-efficient of variation of different measures of coronary 18F-fluoride activity.**

Variation of coronary 18F-fluoride measurements using different anatomical sites for measurement of background tracer activity.

Paired t-tests used compared with TBR_MAX_ (LA): *, p≤0.05, ****, p ≤0.0001.

TBR, target-to-background ratio; BCV, brachiocephalic vein; SVC, superior vena cava; LA, left atrium.


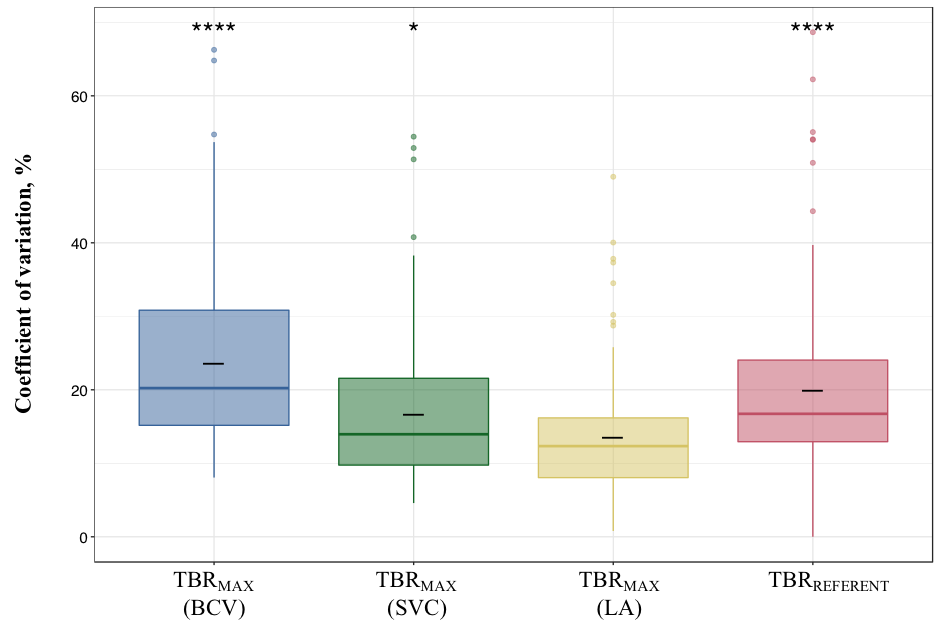


**Supplementary Figure 4.**

**Scan-rescan repeatability of coronary 18F-fluoride uptake.**

Examples of repeated 18F-fluoride positron emission tomography and computed tomography scans across different thresholds of uptake. There were high levels of agreement between scans using a TBR_MAX_ >0.9 (93.0%, TBR_MAX_ >0.9, 94.2%, TBR_MAX_ >1.1).


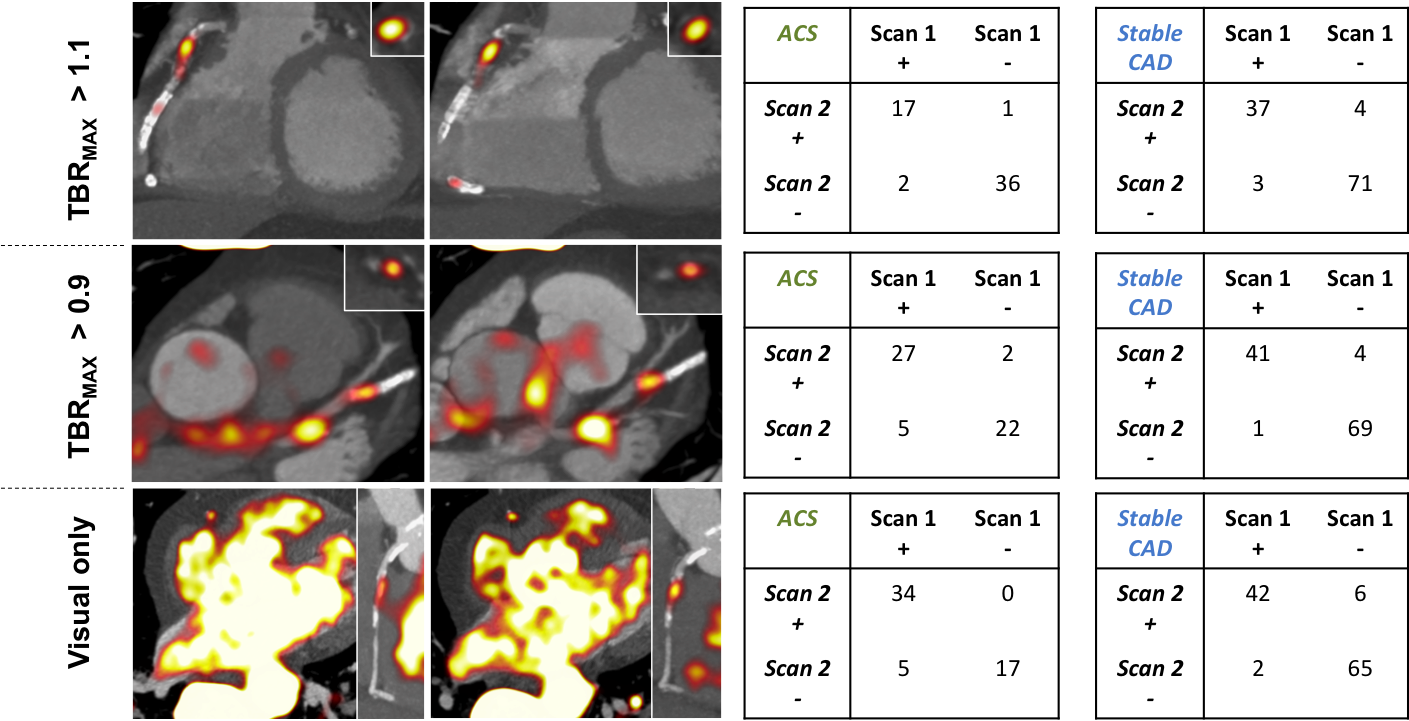


**Supplementary Table 1.**

Bland-Altman analysis of background 18F-fluoride cardiac activity for between observer (A) and between scan (B) reproducibility.

|  | Blood pool Suv_mean_ | | | |
| --- | --- | --- | --- | --- |
| Location |  | DIFFERENCE | |  |
|  | OVERALL MEAN | MEAN | SD | 95% LIMITS OF AGREEMENT |
| (A) |  |  |  |  |
| BRachiocephalic vein | 0.903 | -0.08 | 0.16 | -0.394 to 0.233 |
| Interventricular septum | 0.771 | 0.028 | 0.052 | -0.074 to 0.131 |
| Left atrium | 1.159 | -0.018 | 0.043 | -0.103 to 0.067 |
| Left ventricle | 1.142 | -0.016 | 0.064 | -0.142 to 0.110 |
| Right atrium | 1.13 | -0.009 | 0.053 | -0.112 to 0.095 |
| Right ventricle | 1.124 | -0.019 | 0.051 | -0.118 to 0.080 |
| Superior vena cava | 1.084 | -0.013 | 0.174 | -0.355 to 0.328 |
| (B) |  |  |  |  |
| Brachiocephalic vein | 0.863 | -0.056 | 0.188 | -0.423 to 0.312 |
| Interventricular septum | 0.785 | -0.038 | 0.098 | -0.229 to 0.154 |
| Left atrium | 1.15 | -0.024 | 0.114 | -0.248 to 0.200 |
| Left ventricle | 1.134 | -0.048 | 0.119 | -0.282 to 0.185 |
| Right atrium | 1.126 | -0.029 | 0.131 | -0.286 to 0.227 |
| Right ventricle | 1.114 | -0.022 | 0.134 | -0.284 to 0.240 |
| superior vena cava | 1.078 | -0.049 | 0.17 | -0.382 to 0.285 |

**Supplementary Table 2.**

Scanning variables in (A) patients with myocardial infarction, and (B) stable coronary artery disease.

| (A) | Scan 1 | Scan 2 |
| --- | --- | --- |
| Number | 10 | 10 |
| Heart rate, /min | 63.7±9.1 | 59.6±9.9 |
| Dose length product, mGy | 278±103 | 336±112 |
| 18F-Fluoride dose, MBq | 242±9 | 239±9 |
| Tracer administration to scan, min | 61±4 | 62±3 |

| (B) | scan 1 | scan 2 |
| --- | --- | --- |
| Number | 20 | 20 |
| Heart rate, /min | 56.8±8.0 | 57.3±10.3 |
| Dose length product, mGy | 448±259 | 394±226 |
| 18F-Fluoride dose, MBq | 249±9 | 248±9 |
| Tracer administration to scan, min | 66±7 | 66±10 |

Values are mean±standard deviation.
