# Supplementary figures and images for: Molecular Coronary Plaque Imaging Using 18F-Fluoride
Source: Circ Cardiovasc Imaging. 2019 Aug 6;12(8):e008574. doi: 10.1161/CIRCIMAGING.118.008574 (PMC7668410; doi:10.1161/CIRCIMAGING.118.008574)

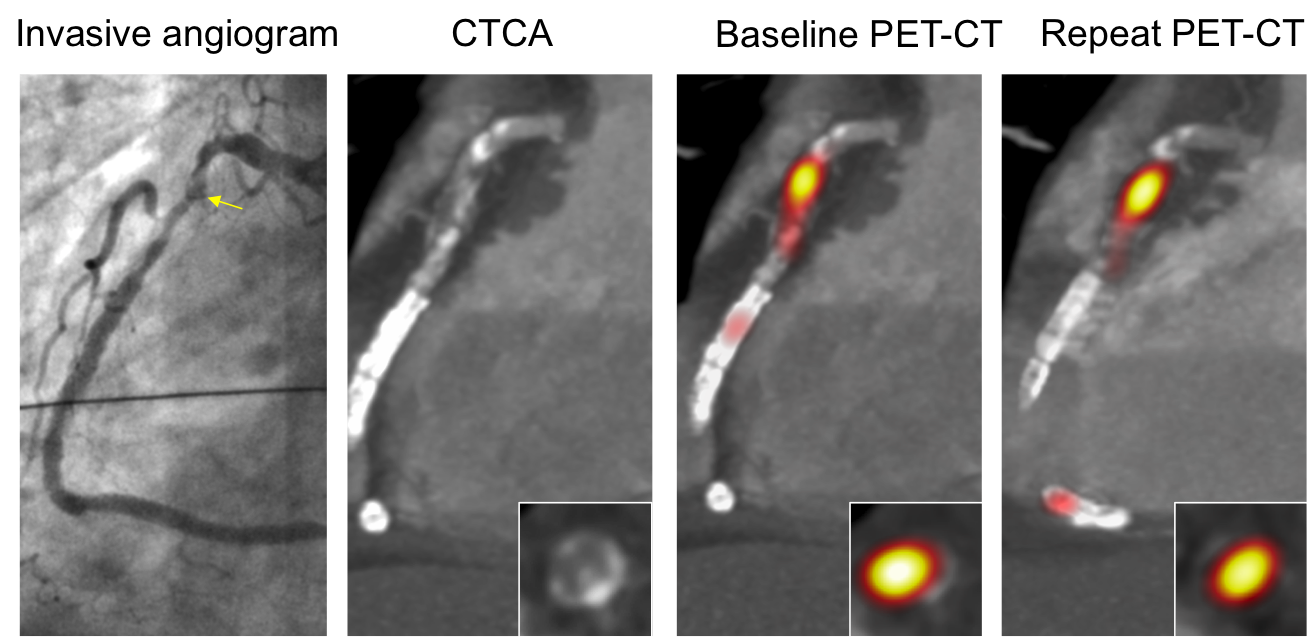

Supplement: Supplementary file 3 [file hci-12-e008574-s003.png]
