# Supplementary material for: Molecular Coronary Plaque Imaging Using 18F-Fluoride
Source: Circ Cardiovasc Imaging. 2019 Aug 6;12(8):e008574. doi: 10.1161/CIRCIMAGING.118.008574 (PMC7668410; doi:10.1161/CIRCIMAGING.118.008574)
Supplement: Supplementary file 2 [file hci-12-e008574-s002.docx]

|  | **EDINBURGH MEDICAL SCHOOL**  **DEANERY of CLINICAL SCIENCES** | |
| --- | --- | --- |
| UNIVERSITY/BHF CENTRE FOR CARDIOVASCULAR SCIENCE | | |
| Dr. R.J. Gropler  Editor-in-Chief  *Circulation: Cardiovascular Imaging*  200 Fifth Avenue  Suite 1020  Waltham, MA 02451  United States of America  18^th^ June 2019 | | Chancellor’s Building  49 Little France Crescent  Edinburgh  EH16 4SB |

Dear Dr. Gropler

**Re:	Molecular coronary plaque imaging using 18F-fluoride**

Thank you for considering our revised manuscript for publication in *Circulation: Cardiovascular Imaging.*

We, the authors, agree to the inclusion of Richard Parker as a co-author on this manuscript.

Yours sincerely,

Dr. Alastair Moss

Dr. Mhairi Doris

Dr. Jack Andrews

Dr. Rong Bing

Dr. Marwa Daghem

Prof. Edwin van Beek

Dr. Laura Forsyth

Dr. Anoop Shah
Dr. Michelle Williams

Dr. Stephanie Sellers

Dr. Jonathon Leipsic

Dr. Marc Dweck

Prof. David Newby

Dr. Philip Adamson
